# Supplementary material for: The time course of molecular acclimation to seawater in a euryhaline fish
Source: Sci Rep. 2021 Sep 13;11:18127. doi: 10.1038/s41598-021-97295-3 (PMC8438076; doi:10.1038/s41598-021-97295-3)
Supplement: Supplementary file 2 — Supplementary Figures. [file 41598_2021_97295_MOESM2_ESM.docx]

**Supplementary figures**

Supplementary Figure 1. Principal component analysis (PCA) of variance stabilized expression values of the 500 most variable genes for *Aphanius dispar* gills at the different sampling events. 22% of the total variation is explained by the first two components. Data ellipse for day 7 samples was not drawn because of the small sample size.


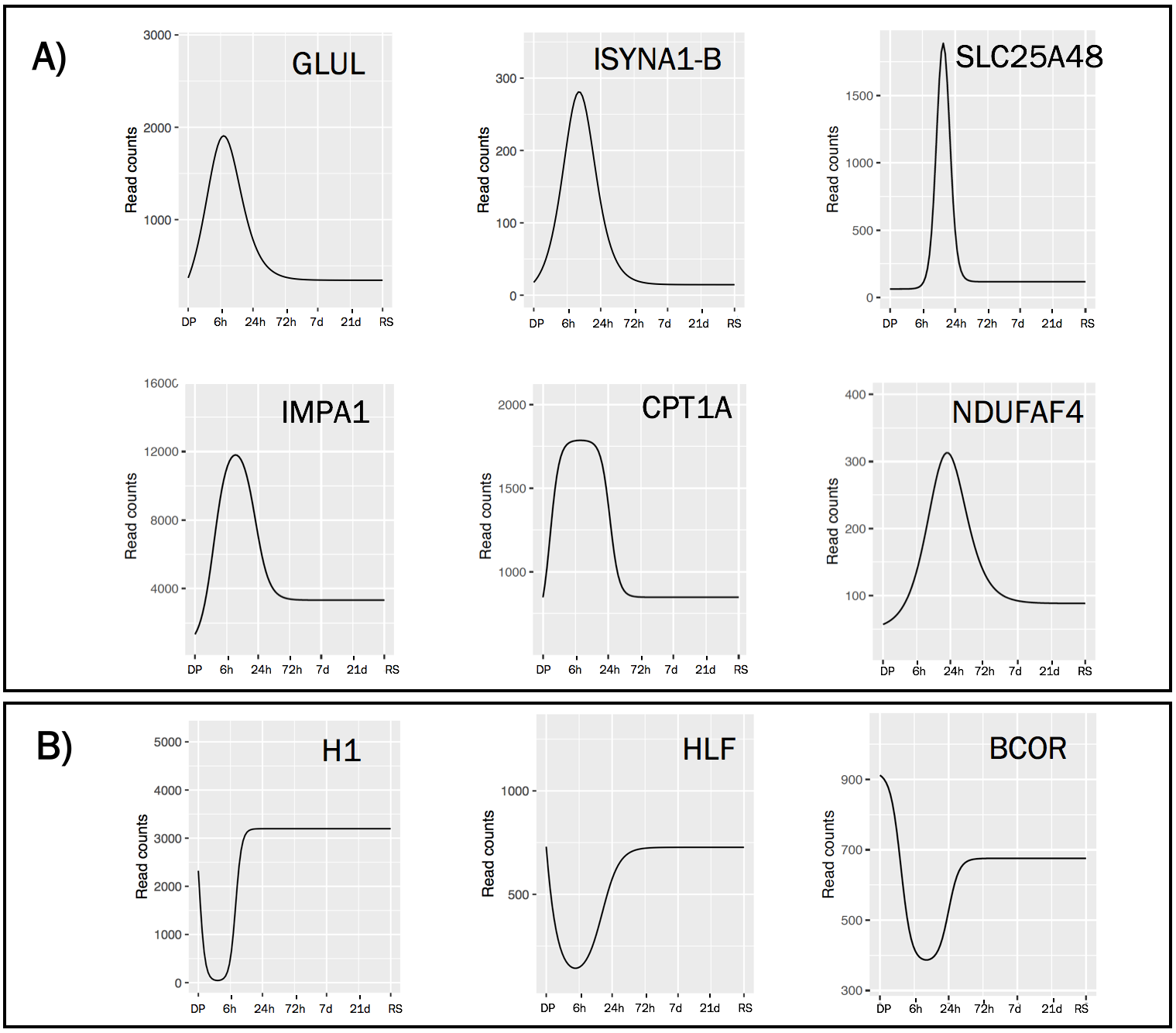


Supplementary Figure 2. Expression profiles of ImpulseDE2 identified transiently upregulated (A) and downregulated (B) genes in the first 24 hours.
